# Delayed graft function after renal transplantation

**DOI:** 10.1515/med-2025-1140

**Published:** 2025-02-12

**Authors:** Darijus Skaudickas, Povilas Lenčiauskas, Augustas Skaudickas, Andrejus Bura

**Affiliations:** Lithuania Medical Academy, Lithuanian University of Health Sciences, Eiveniu 2, 50161, Kaunas, Lithuania; Lithuania Medical Academy, Lithuanian University of Health Sciences, Kaunas, Lithuania

**Keywords:** delayed graft function, kidney transplantation, kidney failure

## Abstract

**Background information:**

Delayed graft function (DGF), defined as the need for dialysis within the first week of a kidney transplant, is a common complication, particularly in extended criteria donor transplants, where its incidence ranges from 21 to 31%.

**Objectives:**

We observed a prolonged case of DGF in a 47-year-old patient with chronic kidney disease (CKD) resulting from diabetic nephropathy. The patient, classified in a moderate immunologic mismatch group, received a marginal deceased donor kidney.

**Results:**

For the first 4 weeks post-transplantation, graft function was impaired. After 29 days of anuria, the transplanted kidney began to recover. The literature review found few clinical cases of DGF extending beyond 1 month. Our patient had several risk factors for DGF, including diabetes mellitus, pre-transplant hemodialysis, and moderate immunologic mismatch. Additionally, the marginal graft increased the risk of ischemia-reperfusion injury and glycocalyx damage. However, it remains unclear how these factors influenced the duration of DGF. The exact cause of the extended DGF in this case remains unknown. Although the literature identifies key risk factors for DGF, data on factors leading to prolonged kidney dysfunction are lacking. Therefore, decisions to remove a non-functioning transplanted kidney should not be made hastily.

## 1 Introduction

Delayed graft function (DGF) is a common term used to describe the initial dysfunction of a transplanted kidney, often due to various perioperative and operative risk factors [1]. These factors can be categorized into donor-related (e.g., organ quality, donor vital signs, age, higher body mass index, acute kidney injury, ethnicity, and shipping distance), recipient-related (e.g., comorbidities, pre-transplant dialysis, HLA mismatch, ABO incompatibility, previous kidney transplants, and higher BMI), and perioperative factors (e.g., hemodynamic instability, calcineurin inhibitors, and nephrotoxic drugs). The primary risk factors contributing to DGF include ischemia-reperfusion injury (IRI), donor kidney origin (deceased or living donation), duration of cold ischemia time (CIT) or warm ischemia time (WIT), and the recipient’s clinical status [2]. DGF is more frequently encountered in recipients of deceased donor kidney transplants, whereas it is relatively rare in living-related renal transplants [3]. The immunological mismatch between the donor and recipient is also a significant contributor to DGF. To mitigate this unfavorable outcome, it is crucial to enhance awareness and conduct comprehensive analyses of renal transplantation procedures globally. Multiple studies have demonstrated that hypothermic machine perfusion (HMP) of deceased donor kidneys, as opposed to cold storage, reduces the incidence of DGF [4]. These findings emphasize the importance of optimizing donor–recipient matching, refining surgical techniques, enhancing postoperative care protocols, and implementing preventive measures to reduce DGF incidence. As DGF is increasingly recognized in contemporary research and clinical settings, further investigation is necessary to gain deeper insights, conserve clinical resources, and fundamentally improve recipients’ quality of life.

## 2 Case presentation

A 47-year-old patient was admitted to our hospital as a candidate for marginal kidney transplantation. At admission, the patient was asymptomatic. His medical history included over 25 years of type 1 diabetes complicated by diabetic nephropathy (diagnosed 3 years prior), polyneuropathy, and retinopathy. He was diagnosed with stage 5 chronic kidney disease (CKD) in December 2019 and was on hemodialysis via an arteriovenous fistula. In January 2022, he was listed for a kidney transplant. The patient had a BMI of 20.22 kg/m^2^ and an arterial blood pressure of 170/105 mmHg in both arms. Immunologic compatibility testing revealed four incompatible antigens placing him at moderate risk of immunological mismatch. Pre-transplant imaging showed no significant stenosis in the iliac arteries. Immunosuppression was induced with basiliximab, methylprednisolone, and mycophenolate mofetil. The kidney, preserved using HMP, was transplanted without surgical complications (CIT was 18 h; WIT was 44 min).

**Figure 1:**
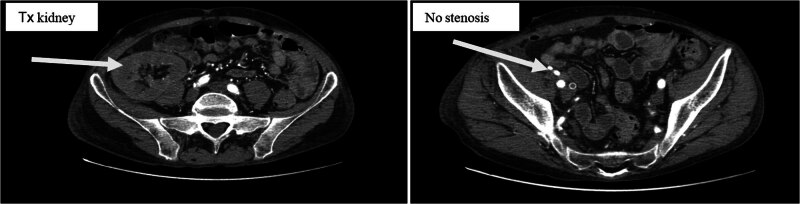
Urgent CT angiography.

**Figure 2:**
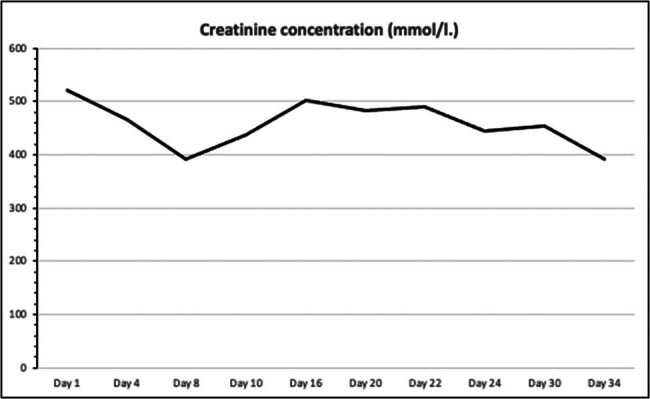
Serum creatinine concentration during the course of treatment.

**Figure 3:**
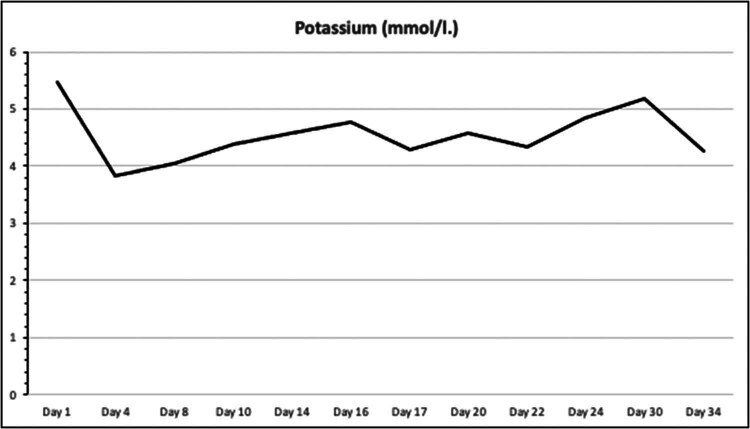
Serum potassium levels during the course of treatment.

**Figure 4:**
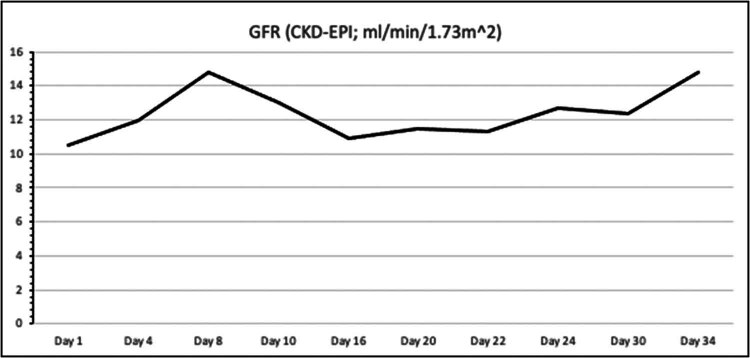
Change in GFR during the course of treatment.


On the first postoperative day, the patient was admitted to the ICU for close monitoring. Despite continued immunosuppression, anuria persisted, and DGF was diagnosed, necessitating continued hemodialysis. By postoperative day 7, the patient remained anuric, with Doppler ultrasound indicating a high resistive index, suggestive of renal artery stenosis. However, CT angiography ruled out stenosis (Figure 1). On postoperative day 16, due to continued graft dysfunction, new anti-donor antibodies were detected, cross-reaction flow cytometry was positive, and a biopsy revealed active antibody-mediated rejection (ABMR) with peritubular leukostasis and acute tubular damage. Treatment included methylprednisolone pulse therapy, plasmapheresis, and intravenous immunoglobulin. By postoperative day 29, the kidney function began to improve, with diuresis exceeding 2,000 ml and normalization of creatinine, urea, and electrolyte levels (Figures 2–4). The patient was discharged for outpatient care. Creatinine and GFR levels returned to normal during the outpatient follow-up period.

**Informed consent:** Written informed consent was obtained from the participant/patient(s) for the publication of this report.

## 3 Discussion

DGF is often described in the literature as the need for dialysis during the first week of kidney transplantation [5]. However, in rare cases, the graft function can remain diminished for extended periods [6]. According to the literature review, we found that prolonged DGF lasting more than 14 days [7] or 28 days [8] negatively affected graft survival during the follow-up period. Reports of DGF lasting more than 1 month are rare. For example, Thye et al. [3] described a case of DGF lasting 132 days in a 47-year-old patient with a living-related kidney transplant. Similarly, Schulz et al. [9] reported a 148-day anuric episode in a 59-year-old recipient of a living-unrelated kidney transplant. In both cases, the kidney function fully recovered. In our case, the kidney function began to improve after 29 days, which is a shorter but still atypical timeframe for DGF. Two days after the initial improvement, the graft regained full function. Several factors could explain this. The age of the donor is a fixed risk factor that elevates the likelihood of DGF and graft failure [10]. This is because the mechanism of cell renewal is reduced, or tolerance to the ischemic state is poor, which may lead to irreversible cell necrosis due to IRI syndrome.

Additionally, Smudla et al. found that DGF was related to lower residual diuresis, higher intradialytic weight gain, and higher fluid balance during the first postoperative day [11]. Unfortunately, we did not analyze this patient’s body hydration status. According to an analysis by Bura et al. at Kaunas University of Health Sciences Hospital in Lithuania, recipients after kidney transplantation in the early pre- and post-transplant period are usually hyperhydrated [12]. Systematic venous congestion and volume overload can decrease kidney perfusion and prolong the ischemic period [13].

IRI is the most critical process occurring during the explantation and transplantation of the kidney graft. It involves complex pathophysiology, including cell death, endothelial dysfunction, transcriptional reprogramming, and activation of the innate and adaptive immune systems. Due to the decrease in oxygen supply, cells switch to anaerobic metabolism, destabilizing lysosomal membranes with leakage of lysosomal enzymes, breakdown of the cytoskeleton, and inhibition of membrane-bound Na^+^/K ± ATPase activity. This process initiates intracellular accumulation of Na^+^ ions and water due to cellular edema [14]. After reperfusion, all processes return to normal, but intracellular hypercalcemia, which is elevated during the ischemic stage, activates calpains, causing injury to the cell structure and cell death. This leads to an uncontrolled release of cellular fragments into the extracellular space [15]. At the vascular level, IRI leads to swelling of endothelial cells, loss of glycocalyx, and degradation of the cytoskeleton. Consequently, intercellular contact between endothelial cells is lost, increasing vascular permeability and fluid loss to the interstitial space. In expanded criteria brain dead donors, pronounced IRI is always present, and the results of early kidney function depend on the severity of IRI and the recipient’s status before kidney transplantation. Therefore, older donors have less intracellular ability to repair after IRI [16].

The second reason could be glycocalyx damage to the transplanted kidney endothelium. A study by Hui Liew aimed to examine the longitudinal effects of kidney transplantation on the glycocalyx by measuring biochemical markers of glycocalyx, endothelial dysfunction, and the perfused boundary region (PBR) through direct visualization of cortical peritubular capillaries in transplanted kidneys. The study found that PBR improved and syndecan-1 levels (a marker of endothelial glycocalyx degradation) decreased after living donor kidney transplantation compared with cadaveric donor kidneys, which suffer from prolonged ischemic time. These findings demonstrate that damage to the endothelial glycocalyx develops early in the course of renal IRI. The combination of reduced PBR and syndecan-1 following transplant suggests that transplantation may improve glycocalyx stability (at 3 months post-transplant) [17]. This study demonstrates that ischemically injured marginal donors’ kidneys are characterized by reduced microvascular blood flow and loss of glycocalyx integrity compared with kidneys from living donors. Unfortunately, we did not find publications comparing changes in glycocalyx biomarkers in patients with different early and DGF. In our case, several factors could have led to DGF. DGF increases the immunogenicity of post-transplant grafts and the risk of rejection and mortality in post-transplant patients. The ABMR was previously loosely correlated with the presence of acute glomerulitis, which is the infiltration of the glomerulus by numerous mononuclear cells [18]. In our case, we detected the donor’s antibody and performed a transplanted kidney biopsy. The relative hazard for biopsy-proven acute rejection in DGF (vs no DGF) was 1.55 [19].

As mentioned previously, DGF is becoming a more common complication among kidney transplant recipients due to the critical shortage of organs and the use of marginal kidneys [20].

It has been proven that kidney function is diminished due to IRI and potential glycocalyx damage in these cases. Kidney transplantation is a complex medical procedure that also depends on various recipient and perioperative factors [21]. One of the risk factors can be immunologic mismatch and sensitization, which has a clinically significant impact on early and long-term graft function [22]. In our case, the patient was evaluated as having a moderate immunologic mismatch risk. However, the literature states that it is not uncommon to transplant a kidney in such a patient group while using adequate immunosuppression therapy [23]. For immunologic induction therapies in moderate-risk kidney transplant patients, there are several options. The first-choice agent for early acute rejection prophylaxis has long been basiliximab, which inhibits IL-2 receptors. Recent studies show that rabbit-derived anti-thymocyte globulin has a greater effect on preventing acute graft rejection in higher-risk patients [24]. Our patient received basiliximab-based induction therapy, along with intravenous methylprednisolone and mycophenolate mofetil.

Another significant risk factor associated with DGF is pre-transplant dialysis. Both the procedure and the duration of kidney replacement therapy have a significant impact on short- and long-term graft functions. A few decades ago, pre-transplant dialysis was certainly perceived as a clear risk factor. However, recent literature calls for a reconsideration of this view. Some articles state that hemodialysis has no negative effect on graft survival and function, while peritoneal dialysis results in an even better post-transplant survival rate [25]. Our patient had been treated with hemodialysis for 2.5 years prior to transplantation, which should not place him at additional risk for DGF. Finally, diabetes mellitus has recently been identified as a risk factor for DGF [26]. The precise mechanism of this effect is not yet fully understood, but poor blood glucose control leads to a greater chance of transplanted kidney tissue damage.

Apart from analyzing factors that could have caused this extended DGF condition, clinical management of these patients is equally challenging. Since the expanded criteria donors’ kidney cells have significantly low repair and recovery mechanisms, this is an unshakable factor for DGF. Fortunately, there are new methods to avoid DGF from expanded criteria donors.

The function of the allograft also depends on the conditions of preservation after explantation. Static cold storage (SCS) and HMP are two primary options for renal allograft preservation. Compared with SCS, HMP decreases the incidence of DGF and protects the graft function. However, more evidence is still needed to prove the advantages of HMP. In our case, we used HMP with normal perfusion parameters. Randomized controlled trials comparing the effect of HMP and SCS in deceased donor kidney transplantation were identified through searches of the MEDLINE, EMBASE, and Cochrane databases between January 1, 1980 and December 30, 2017. The results indicated that compared with SCS, HMP decreased the incidence of DGF (RR: 0.78, 95% CI: 0.69–0.87, *P* < 0.0001) and improved graft survival at 3 years (RR: 1.06, 95% CI: 1.02–1.11, *P* = 0.009) [27]. Despite our clinical case study, the use of HMP resulted in DGF. We believe that impaired allograft function had several etiological and pathophysiological factors that contributed to the clinical status.

One promising tool in kidney transplantation is normothermic machine perfusion (NMP), which could potentially improve the outcome of expanded criteria donor kidney transplantation and reduce the DGF ratio [28,29]. Future double-blinded, randomized, large clinical trials will be required to evaluate the benefits of NMP technology [30].

Choosing adequate immunosuppressive therapy to prevent graft rejection while maintaining a certain immunity status to avoid opportunistic infections is also important. Another dilemma that physicians face in these types of cases is deciding whether to remove a transplanted kidney. Over time, a non-functioning graft can become a source of infection and a stimulus for a systemic immune response, thus becoming more of a problem than a solution. In our case, the patient did not experience graft infection or develop a systemic immune response; therefore, despite not functioning, the kidney was not removed. In our opinion, there is no need to rush to perform a transplantectomy unless there are absolute indications for DGF and a high risk of infection.

Despite DGF being a common complication of kidney transplantation, in our case, it lasted up to 29 days, which is highly unusual for this condition. The patient did have several risk factors for DGF that could have impacted the transplanted kidney’s function, but there are no data in the current literature indicating that these aspects could affect the duration of the post-transplant anuric period. It is still not clear whether the listed risk factors manifested the condition independently or if an interplay between certain conditions led to this type of reaction. Furthermore, this case could serve as a valuable example for further analyzing the use of immunosuppressive medications in moderate-to-high immunologic risk kidney transplant patients, as well as treatment modalities for acute rejection reactions.

## 4 Conclusions

We report a highly extended DGF in a 47-year-old patient with diabetes mellitus and CKD, with kidney function fully recovering after 29 days of anuria. The case highlights the complexity of DGF, which may result from an interplay of donor, recipient, and perioperative factors. Our findings suggest that extended DGF, while rare, is possible, and further research is needed to understand its etiology and management. This case underscores the importance of individualized treatment strategies in kidney transplantation, particularly in patients with moderate-to-high immunologic risk.
